# Supplementary material for: Complex‐centric proteome profiling by SEC‐SWATH‐MS
Source: Mol Syst Biol. 2019 Jan 14;15(1):e8438. doi: 10.15252/msb.20188438 (PMC6346213; doi:10.15252/msb.20188438)
Supplement: Supplementary file 8 — Dataset EV7 [file MSB-15-e8438-s008.zip › feature_plots_string/P26010.pdf]

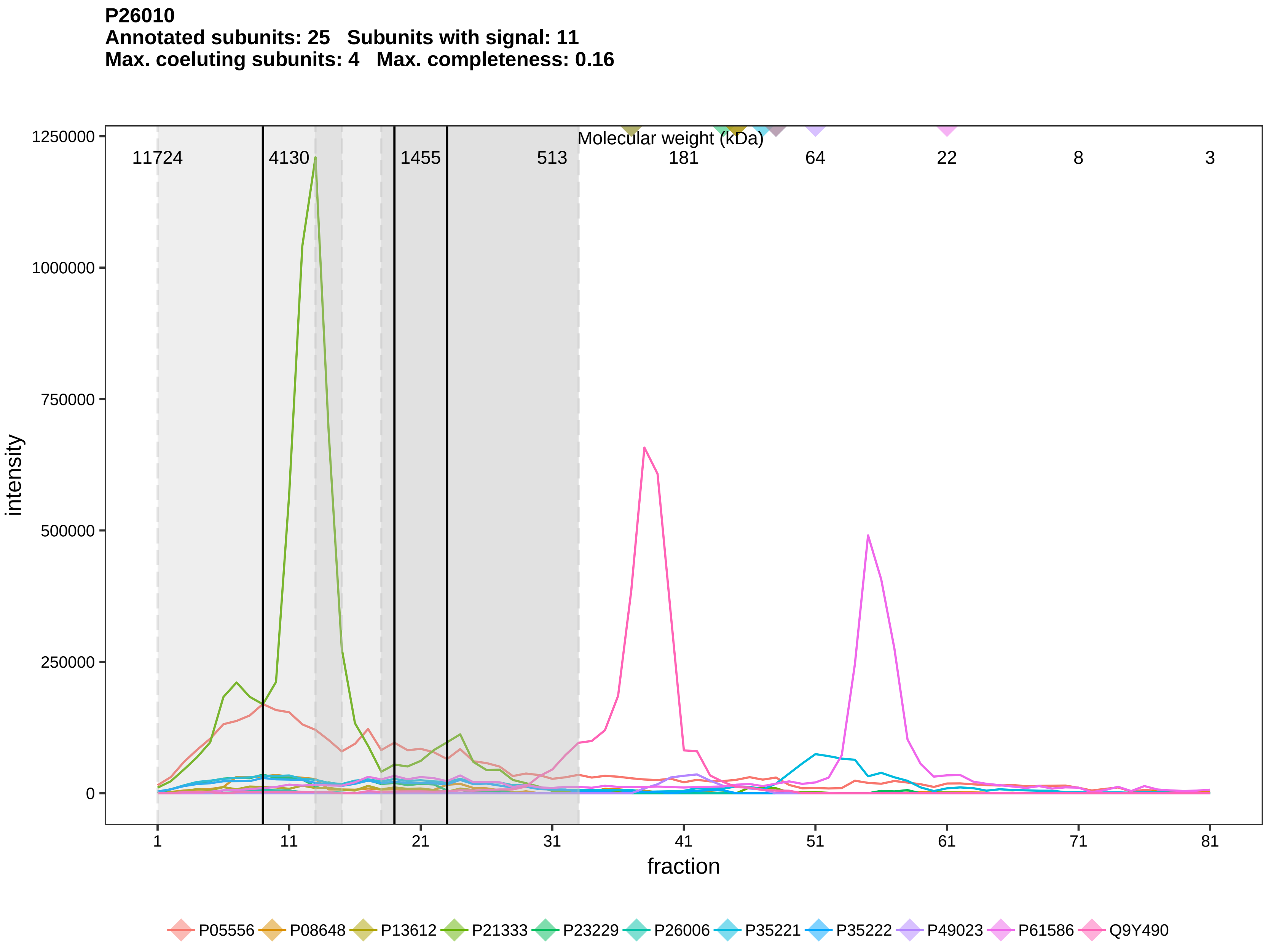
P26010
Annotated subunits: 25 Subunits with signal: 11
Max. coeluting subunits: 4 Max. completeness: 0.16
Molecular weight (kDa)
11724
4130
1455
513
181
64
22
8
3
1250000
1000000
750000
500000
250000
0
intensity
1
11
21
31
41
51
61
71
81
fraction
P05556
P08648
P13612
P21333
P23229
P26006
P35221
P35222
P49023
P61586
Q9Y490
